# Bridging agro-science and human nutrition: zinc nanoparticles and biochar as catalysts for enhanced crop productivity and biofortification

**DOI:** 10.3389/fpls.2024.1435086

**Published:** 2024-08-16

**Authors:** Nazir Ahmed, Lifang Deng, Mehar-un-Nisa Narejo, Iqra Baloch, Lansheng Deng, Sadaruddin Chachar, Yongquan Li, Juan Li, Bilquees Bozdar, Zaid Chachar, Faisal Hayat, Muzafaruddin Chachar, Lin Gong, Panfeng Tu

**Affiliations:** ^1^ College of Horticulture and Landscape Architecture, Zhongkai University of Agriculture and Engineering, Guangzhou, Guangdong, China; ^2^ Institute of Biomass Engineering, South China Agricultural University, Guangzhou, China; ^3^ Faculty of Crop Production, Sindh Agriculture University, Tandojam, Pakistan; ^4^ College of Natural Resources and Environment, South China Agricultural University, Guangzhou, China; ^5^ College of Agriculture and Biology, Zhongkai University of Agriculture and Engineering, Guangzhou, Guangdong, China; ^6^ Dongguan Yixiang Liquid Fertilizer Co. Ltd., Dongguan, China

**Keywords:** shelf life, post-harvest, biofortification, abiotic stress tolerance, human health, microbial facilitation, Zn deficiency

## Abstract

The integration of zinc nanoparticles (Zn NPs) with biochar offers a transformative approach to sustainable agriculture by enhancing plant productivity and human nutrition. This combination improves soil health, optimizes nutrient uptake, and increases resilience to environmental stressors, leading to superior crop performance. Our literature review shows that combining Zn NPs with biochar significantly boosts the crop nutrient composition, including proteins, vitamins, sugars, and secondary metabolites. This enhancement improves the plant tolerance to environmental challenges, crop quality, and shelf life. This technique addresses the global issue of Zn deficiency by biofortifying food crops with increased Zn levels, such as mung beans, lettuce, tomatoes, wheat, maize, rice, citrus, apples, and microgreens. Additionally, Zn NPs and biochar improve soil properties by enhancing water retention, cation exchange capacity (CEC), and microbial activity, making soils more fertile and productive. The porous structure of biochar facilitates the slow and sustained release of Zn, ensuring its bioavailability over extended periods and reducing the need for frequent fertilizer applications. This synergy promotes sustainable agricultural practices and reduces the environmental footprint of the traditional farming methods. However, potential ecological risks such as biomagnification, nanoparticle accumulation, and toxicity require careful consideration. Comprehensive risk assessments and management strategies are essential to ensure that agricultural benefits do not compromise the environmental or human health. Future research should focus on sustainable practices for deploying Zn NPs in agriculture, balancing food security and ecological integrity and positioning this approach as a viable solution for nutrient-efficient and sustainable agriculture.

## Graphical Abstract

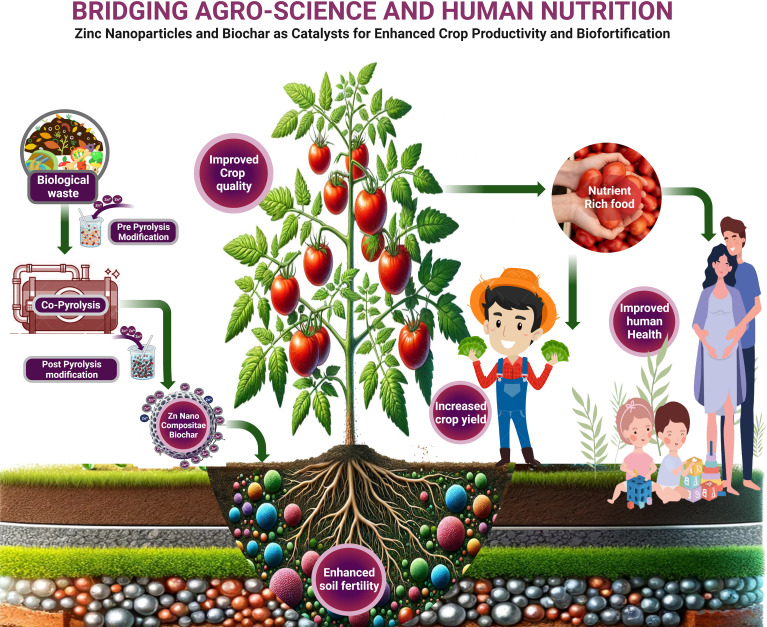

## 1 Introduction

Zinc (Zn) is an essential trace element that plays a crucial role in various biological processes in both plant and animal kingdoms. It is a key component of over 300 enzymes that facilitate critical functions, such as cell division, DNA synthesis, and glucose metabolism, underpinning cellular activities (Balandrán-Valladares et al., 2021; Ahmed et al., 2024b). In plants, Zn is fundamental to numerous metabolic pathways that influence photosynthesis, growth regulation, and the synthesis of indole-3-acetic acid, a plant hormone that regulates cell elongation, root branching, and flowering (Balafrej et al., 2020; Tripathi et al., 2022; Zhao et al., 2024). Consequently, Zn deficiency can lead to significant disruptions in plant growth and development, manifested as stunted growth, chlorosis, and reduced crop yield and quality (Suganya et al., 2020; Ahmed et al., 2024b). Almost half of the world’s arable soils are deficient in Zn, which poses a serious threat to global agriculture. This deficiency affects an estimated 2 billion people who rely on crops grown in these deficient soils (Waqeel and Khan, 2021; Khan et al., 2022). Widespread deficiency is particularly prevalent in regions with calcareous soils and high pH levels, where Zn availability is limited (Jalal et al., 2020). Additionally, organic matter can bind Zn, making it less available for plant uptake, which poses a challenge in soils with high organic content. Moreover, excessive use of phosphate fertilizers can reduce Zn availability to plants (Alloway, 2008; Suganya et al., 2020; Saboor et al., 2021), causing a potential reduction in crop yield ranging from 20 to 40% depending on the crop and severity of the deficiency (Choudhary et al., 2019; Saboor et al., 2021). This not only threatens food security, but also exacerbates nutritional deficiencies in human populations (Garcia-Oliveira et al., 2018; Khan et al., 2022).

**Figure 1:**
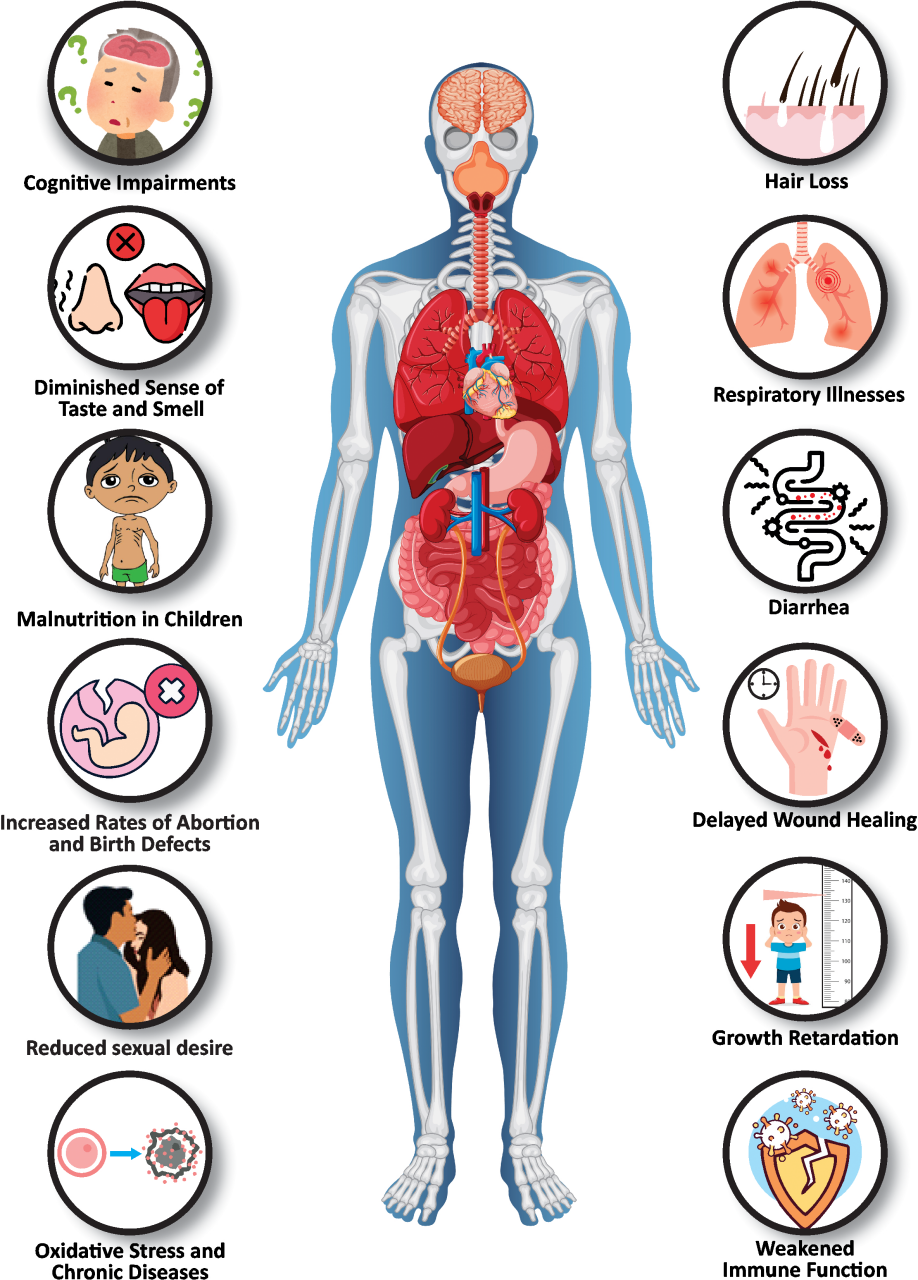
The spectrum of Zn deficiency symptoms in humans. Zn deficiency can lead to cognitive impairments, loss of taste and smell, hair loss, increased susceptibility to respiratory illnesses, and gastrointestinal issues like diarrhea. In children, it causes malnutrition and growth retardation. Reproductive health effects include increased rates of abortion, birth defects, and reduced sexual desire. Chronic diseases may arise due to oxidative stress and weakened immune function.

According to the World Health Organization (WHO), nearly 800,000 deaths annually are attributed to Zn deficiency, with a substantial number occurring among children under five (Arfi et al., 2021; Waqeel and Khan, 2021). This widespread deficiency leads to severe health problems such as stunted growth, weakened immune function, and severe neural developmental disorders (**Figure 1**). The prevalence of Zn deficiency is particularly high in areas where cereal-based diets that are low in bioavailable Zn are predominant (Prasad, 2013; Rehman et al., 2020; Younas et al., 2023). Its deficiency can impair immune function, increase susceptibility to infections, delay wound healing, and, in severe cases, cause growth retardation and cognitive impairment in children (Bhatnagar and Taneja, 2001; Hussain et al., 2022). Zn is essential during vulnerable stages such as pregnancy, infancy, and childhood, not only to strengthen immunity but also to support neurological development and safeguard against infections (Vickram et al., 2021). It plays a critical role in reproductive health, influencing the development of reproductive organs and hormone regulation, with deficiencies linked to conditions such as hypogonadism and adverse pregnancy outcomes, including an increased risk of abortion and birth defects (Durrani and Parveen, 2021). Zn is also important for tissue repairs it is a key factor in collagen synthesis, cellular proliferation, and management of the inflammatory response, which are vital for effective wound healing (Chen et al., 2022; Molenda and Kolmas, 2023). Additionally, Zn deficiency can impair taste and smell, leading to hypogeusia and hyposmia, and contribute to hair loss owing to its role in follicular cell proliferation (Cummings and Kovacic, 2009; Trüeb, 2020). A Zn shortfall can diminish antiviral immunity, making individuals more susceptible to viral infections such as herpes, hepatitis C, HIV, and COVID-19 (Skalny et al., 2020; Samad et al., 2021; Khan et al., 2022). Therefore, addressing Zn deficiencies in both plants and humans is critical for improving global health and food security.

Correcting Zn deficiency is challenging because of several factors, including dietary patterns, environmental challenges, high costs, and the limited effectiveness of chemical Zn fertilizers. Developing Zn-rich crops through plant breeding and genetic engineering is difficult and compounded by genetic variation and the need for substantial financial and technological resources (Khan et al., 2022). Soil properties further complicate this issue by limiting Zn solubility and mobility, which leads to poor plant uptake (Alloway, 2008; Suganya et al., 2020). High soil pH and phosphate can form insoluble Zn compounds, thereby reducing their availability (Rehman et al., 2020; Zulfiqar et al., 2020). Organic matter can also bind Zn, making it less available in soils with a high organic content (Suganya et al., 2020). Conventional breeding methods struggle because of the lack of genetic diversity in the staple crops (Chachar et al., 2015; Garcia-Oliveira et al., 2018). Additionally, genetically modified organisms face regulatory hurdles and public acceptance issues that impede the adoption of Zn-biofortified crops (Prasad, 2013). This multifaceted challenge underscores the need for innovative solutions to effectively address Zn deficiency.

In such scenarios, integrating Zn NPs with biochar can help mitigate these challenges by improving Zn solubility and uptake by plants, showing promise in enhancing the Zn content of crops, thereby addressing Zn deficiencies in human diets (García-López et al., 2019; Younas et al., 2023; Ahmed et al., 2024a). Zn NPs have gained attention because of their ability to enhance nutrient uptake and bioavailability, thereby improving crop yield and quality (Akhtar et al., 2022; Taqdees et al., 2022). Biochar, a carbon-rich product derived from the pyrolysis of organic materials, is known for its soil amendment properties including improved water retention, CEC, and microbial activity (Ahmed et al., 2024a). The high surface area and porous structure of biochar make it an excellent carrier for Zn NPs, facilitating the slow and sustained release of Zn, thereby enhancing its bioavailability over extended periods (Tan et al., 2022; Roy et al., 2022a). Together, Zn NPs and biochar offer a synergistic solution that not only enhances crop productivity, but also contributes to sustainable agricultural practices. However, the introduction of Zn NPs into the agricultural ecosystem is not without risks (Reed et al., 2012; Xiao et al., 2022). There are concerns regarding the long-term stability of Zn NPs in soil and their potential environmental impacts, including biomagnification through the food chain (García-Gómez et al., 2020; Ruiz-Torres et al., 2021). Most current studies are limited by short-term laboratory experiments that do not fully capture the long-term interactions between Zn NPs and biochar under diverse field conditions (Hazeem, 2022; Zhao et al., 2023). Additionally, comprehensive studies on the potential toxicity and environmental risks associated with Zn NPs accumulation in soil are lacking (Shemawar et al., 2021; Hazeem, 2022). This presents a significant gap in our understanding of the long-term implications of Zn NPs in agriculture.

To address the existing gaps, this review explores the synergistic potential of Zn NPs and biochar, focusing on their ability to enhance nutrient uptake, improve crop yields, and offer sustainable agricultural solutions (Jalal et al., 2022). Traditional methods, such as the application of chemical Zn fertilizers, foliar sprays, soil amendments, and crop rotation, often fail to effectively resolve Zn deficiencies because of poor Zn solubility and mobility in the soil (Alloway, 2008; Jalal et al., 2022; Visha Kumari et al., 2022). Additionally, organic matter application and liming are used to improve soil fertility, but they have limited success in addressing Zn deficiencies (Alloway, 2008; Suganya et al., 2020; Saboor et al., 2021). This innovative approach of integrating Zn NPs with biochar is a compelling alternative. Zn NPs increase nutrient bioavailability, whereas biochar enhances soil properties such as water retention and microbial activity (Akhtar et al., 2022; Taqdees et al., 2022). This review critically evaluates the benefits and risks of integrating Zn NPs with biochar, identifies research gaps, and proposes future research directions. It emphasizes the need for long-term field trials across various crops and climates to develop guidelines that maximize agricultural benefits while minimizing environmental risks. Additionally, the potential cost-saving benefits for farmers are discussed, advocating for comprehensive cost-benefit analyses to inform policy decisions and promote the adoption of Zn NPs and biochar in agriculture to enhance food security and sustainability.

## 2 Review methodology

This review synthesizes the current state of knowledge regarding the integration of Zn NPs with biochar and its potential to transform sustainable agricultural practices while addressing Zn deficiencies in humans. To ensure a comprehensive and balanced overview, a systematic approach was employed to gather relevant literature. Databases, including PubMed, Web of Science, Scopus, and Google Scholar, were searched for peer-reviewed articles, reviews, and reports published from 2000 to 2024. The keywords used in the search included Zn NPs, biochar, Zn deficiency, sustainable agriculture, crop yield,” abiotic stress, Zn toxicity, and bioavailability. The inclusion criteria included studies that focused on the use of Zn Bulk, Zn NPs, and biochar in agricultural contexts, their impact on crop yield and quality, and their implications for human health. The exclusion criteria were non-peer-reviewed articles, studies not related to Zn NPs or biochar, and studies published before 2000, unless they were seminal works in the field. Additionally, the review considered relevant conference proceedings, government reports, and guidelines from organizations such as the Food and Agriculture Organization and the WHO. Cross-referencing and Google Scholar were used to identify additional studies that were cited in the selected papers. This methodological approach ensured a thorough and up-to-date understanding of the topic, facilitated identification of research gaps, and proposed future research directions. The literature review also included a critical evaluation of the methodologies and findings of the selected studies to provide a balanced perspective on the benefits and potential risks associated with the integration of Zn NPs and biochar in agricultural practices.

## 3 Zn in bulk versus nano: diverse behavior and applications

Zn in its bulk and nanoparticle forms exhibits distinctly diverse behaviors and applications, which have been brought to the forefront by advances in nanotechnology. Bulk Zn is recognized for its macroscopic qualities stemming from its atomic composition, which yields stable and predictable properties. Zn NPs are distinguished by their microscale dimensions, which impart unique physicochemical properties. These differences are not merely nuances; they are transformative in their respective application domains, particularly in agriculture and industry (Mahdieh et al., 2018; Dimkpa et al., 2019). The high surface-to-volume ratio of Zn NPs enhances their reactivity and bioavailability compared to bulk Zn, making them efficient nano fertilizers. This increased reactivity allows for better nutrient absorption and utilization by plants. Studies, such as those by Mahdieh et al. (2018) demonstrate that Zn NPs significantly improve vegetative growth and yield in pinto bean cultivars, outperforming conventional Zn fertilizers. Similarly, research by Dimkpa et al. (2019) showed that Zn NPs can enhance the growth and yield of wheat by improving Zn uptake and reducing Zn deficiency symptoms more effectively than traditional Zn fertilizers. These implications are significant, as seen in Li et al. (2023), where crops under drought stress responded favorably to Zn NPs, revealing the potential of these particles to bolster growth and yield under adverse conditions. Bulk Zn is crucial for industrial processes such as galvanization, alloy formation, and battery manufacturing, where it plays a vital role in steel recycling and rechargeable Zn-based batteries (Shi et al., 2020; Kania and Saternus, 2023). However, Zn NPs are pioneering new frontiers in healthcare, demonstrating efficacy in drug delivery systems, UV-shielding properties in sunscreens, and antimicrobial capabilities (Chinnapaiyan et al., 2022; Alaryani et al., 2023). However, the excitement around Zn NPs necessitates an in-depth understanding of their environmental impact. Their enhanced reactivity and mobility present unique environmental challenges compared to bulk Zn, emphasizing the need for thorough research on their environmental footprint (Reed et al., 2012; Sorahinobar et al., 2023). This transition from bulk Zn to nanoparticles marks a significant advancement in agriculture, industry, and medicine. While Zn NPs offer considerable benefits, responsible use and environmental risk mitigation are essential to fully realize their potential.

## 4 Role of biochar in soil health and plant growth

Biochar, an organic material formed during the thermochemical decomposition of biomass, has garnered attention because of its potential to enhance soil health and plant growth. Its unique physicochemical properties allow it to serve as a soil amendment, with multifaceted benefits. Biochar is widely recognized for its role in amending soil pH, particularly in the transformation of acidic soils to a more neutral, plant-friendly state (Tan et al., 2021; Tu et al., 2023). This is largely due to the inherently negative surface charge of biochar, which allows it to retain positively charged ions such as Ca²^+^, Na^+^, K^+^, and Mg²^+^. Beyond its direct interaction with soil pH, biochar addition to the soil also enhances CEC, which is crucial for nutrient retention (Lago et al., 2021; Ahmed et al., 2023a). With a higher CEC, essential nutrients, such as Ca, K, and Mg, are held in the soil more effectively, reducing leaching and promoting plant availability. Supporting this, research by Arwenyo et al. (2023) specifically highlighted the significant role of Ca²^+^ in biochar. This study indicates that Ca²^+^ not only contributes to soil alkalinity, but also serves as a major factor in the soil’s buffering capacity, more so than Mg²^+^ and K^+^, thus underscoring the critical influence of Ca²^+^ within the biochar’s composition for soil improvement. The porous structure of biochar provided an excellent matrix for Zn NPs, facilitating the slow and sustained release of Zn (Das and Ghosh, 2023). The extended availability of Zn ensures a steady supply to plants, thereby improving their uptake and utilization (Tan et al., 2022; Roy et al., 2022a). Additionally, biochar can adsorb and immobilize phytate, a major form of organic P that binds to Zn, making it unavailable to plants. By reducing phytate levels, biochar can enhance the bioavailability of Zn, which is crucial for improving the nutritional quality of food crops and addressing Zn deficiency in humans (Abbas et al., 2021; Li et al., 2021).

Moreover, the porous nature of biochar enhances moisture retention in the soil. Gondim et al. (2018) showed that biochar derived from the Caatinga biome species exhibited superior water-holding capacity compared to cashew wood biochar. This ability to retain water can be particularly beneficial in drought-prone regions by offering plants a consistent water supply and fostering resilience during dry spells (Malik et al., 2022). The interaction of biochar with the soil ecosystem extends to the microscopic level. Biochar can stimulate microbial activity and foster a biodiverse and robust microbial community in soil. This thriving microbial environment can promote the decomposition of organic matter, leading to increased nutrient availability and enhanced plant growth (Tan et al., 2022). Synergy between biochar and soil health is prominent during root development. Soils enriched with biochar tend to encourage improved root growth and branching, which is attributed to enhanced soil structure and nutrient availability. This, in turn, results in optimized nutrient and water uptake, ultimately leading to increased plant vitality and yield (Wen et al., 2022). Biochar is a powerful tool for improving soil health and increasing plant growth. Its capability underscores its potential for sustainable agriculture, pointing towards a bright trajectory for ongoing research and practical applications.

## 5 Mechanisms facilitating nutrient delivery via biochar

The promise of biochar in sustainable agriculture is not limited to its direct effects on soil physical and chemical properties but extends to its role in facilitating efficient nutrient delivery. Central to this is the unique adsorption-desorption dynamics inherent to biochar. Its high surface area, laden with functional groups, allows biochar to effectively adsorb essential plant nutrients, thereby acting as a nutrient reservoir (Gwenzi et al., 2018; Elkhlifi et al., 2023). Over time, these adsorbed nutrients are desorbed and made available to plants via complex physical and chemical processes. This regulated release ensures that nutrients are not lost to leaching and are available for plants over an extended period, thereby reducing the frequency of fertilizer application. Furthermore, the presence of biochar in soil creates a synergistic relationship with soil microorganisms, primarily those involved in nutrient cycling (Tan et al., 2022), and provides a habitat for these microorganisms, protecting them from predation and extreme environmental conditions. In return, these microorganisms facilitate the breakdown of organic matter and release of nutrients bound to the biochar, further aiding nutrient delivery to plants (Peng et al., 2023). Finally, the potential of biochar as a vehicle for slow-release fertilizers cannot be overlooked. The porosity of biochar allows it to be infused with liquid fertilizers, which are then slowly released as the soil moisture changes, ensuring a consistent supply of nutrients to plants (Gwenzi et al., 2018). This method not only extends the duration of nutrient availability but also enhances nutrient use efficiency, as a more significant portion of the applied nutrients is taken up by plants rather than lost to the environment.

## 6 Global prevalence of Zn deficiency in soils

Globally, Zn deficiency is a critical concern, affecting nearly 17% of the world’s population, with significant health implications, such as stunted growth, immune dysfunction, and increased vulnerability to diseases such as COVID-19 (Khan et al., 2022). Despite its paramount importance, a significant proportion of agricultural soils worldwide are plagued by Zn deficiency (Galindo et al., 2021). This hinders plant growth and, by extension, reduces crop yield. Zn deficiency in soils is especially pronounced in regions with calcareous soils or elevated pH levels, often resulting from practices, such as leveling fields for uniform irrigation. Mechanical leveling often leads to the removal of topsoil and organic matter that are typically rich in essential micronutrients (Alloway, 2008). This issue is especially acute in parts of South Asia, sub-Saharan Africa, some areas of Latin America, and distinct regions of Australia. In the latter, regions such as southern Australia, Victoria, Queensland, New South Wales, and Western Australia have shown Zn deficiencies in their soils. Notably, Southwest Australia has been identified as the most extensive continuous Zn-deficient area on a global scale, thereby making Australia a pivotal center for micronutrient research (Alloway, 2008; Sadeghzadeh, 2013). Several contributing factors have been identified for the decline in soil Zn levels. Predominantly, repeated and exhaustive cultivation of land without sufficient Zn replenishment has emerged as a primary cause (Sadeghzadeh, 2013; Jalal et al., 2024a). Additionally, increased soil erosion, adoption of high-yielding crop varieties that extract a larger quantity of nutrients, and certain soil management techniques can exacerbate this deficiency (Jalal et al., 2024b). Practices such as liming can further aggravate this situation by increasing the soil pH, thus making Zn less accessible to plants (Alloway, 2008). Addressing this issue requires a comprehensive approach, including soil analysis, targeted Zn amendments such as fertilizers, and biofortification of key crops to enhance Zn content and meet the nutritional needs of the growing global population (Noulas et al., 2018; Zulfiqar et al., 2021b).

## 7 Impact of Zn on agriculture: crop yield and quality

**Figure 2:**
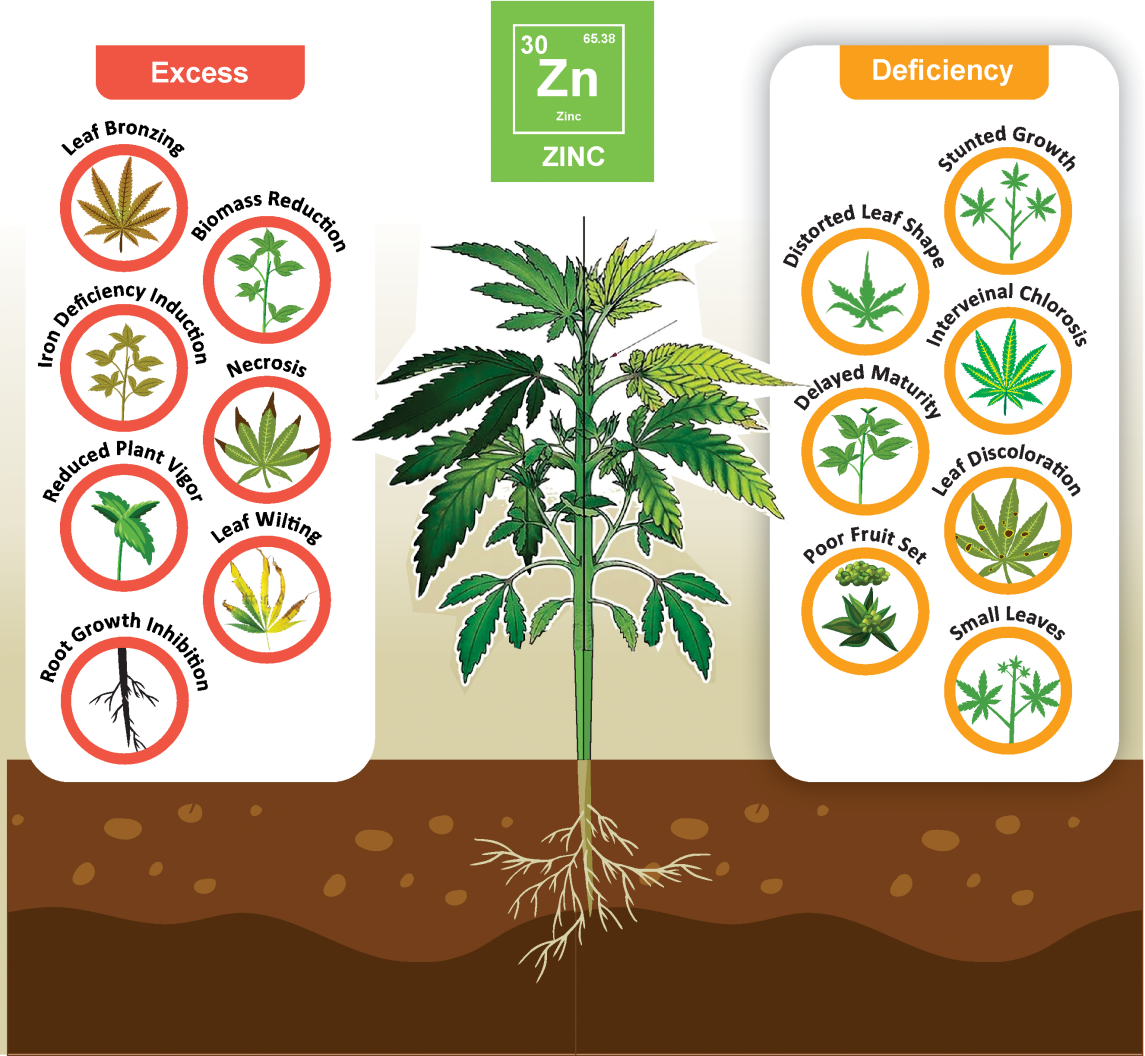
Effects of Zn imbalance in plant health: excess and deficiency symptoms: Diagram illustrates the symptoms associated with zinc imbalance in plants. Excess zinc results in leaf bronzing, biomass reduction, iron deficiency, necrosis, reduced vigor, wilting, and root growth inhibition (Left). Conversely, zinc deficiency causes stunted growth, distorted leaves, interveinal chlorosis, delayed maturity, leaf discoloration, poor fruit set, and smaller leaves (Right).

Zn occupies a central position in plant physiological and biochemical pathways, and its intrinsic roles influence cellular and molecular processes. At the granular level, Zn is integrated into the structural and functional matrices of many enzymes, growth regulators, and proteins (Jalal et al., 2023). Such incorporation ensures the seamless progression of vital physiological functions, including protein synthesis, cellular energy dynamics, and nuanced regulation of gene expression (Noulas et al., 2018). However, when plants are exposed to Zn deficiency, these critical processes can cause significant disturbances. The impact of these disruptions is readily apparent in the form of stunted plant growth, marked chlorosis, and reduction in leaf biomass (**Figure 2**), all of which collectively led to diminished crop yields (Ahmed et al., 2024b). Zn deficiency not only leads to reduced crop yields but also adversely affects the quality of agricultural output (Zhao et al., 2023). Plants with insufficient Zn often produce smaller grains and fruits, suffer from weakened structural integrity, and show an alarming decrease in their micronutrient content, which is essential for their nutritional value and human health (Ishfaq et al., 2021; Ahmed et al., 2024b). Such a nutritional decrement does not merely present a health exigency for human consumers, but also introduces logistical intricacies. Specifically, nutritionally compromised produce is more susceptible to biotic stresses, thereby exacerbating post-harvest storage and logistical challenges.

Upon closer examination, it is clear that various crops display distinct sensitivity to Zn availability. For example, wheat, pepper, citrus, mango, apple, pecan, and lettuce thrive under Zn-rich conditions and exhibit improved characteristics. These enhancements were evident in the form of robust root systems, extended shoot growth, increased vitality, superior fruit quality, grain yield, and biosynthesis of bioactive compounds. Additionally, these plants show increased resistance to biotic and abiotic stresses and represent an extended post-harvest shelf life (Nasir et al., 2016; Zagzog and Gad, 2017; Olechnowicz et al., 2018; Tayyiba et al., 2021; Rehman et al., 2022; de Lima et al., 2023). Under Zn-deficient conditions, certain plant species exhibit distinct developmental anomalies, such as shortened internodes and overall stunted growth, highlighting the detrimental effects of Zn scarcity (Zagzog and Gad, 2017; Meriño-Gergichevich et al., 2021). Farmers experiencing immediate yield losses due to Zn deficiency face financial challenges, which affect economies that are dependent on agriculture on a broader scale. This deficiency jeopardizes food security and nutritional adequacy, leading to higher healthcare costs from malnutrition-related diseases and reduced labor productivity. Consequently, Zn supplementation strategies are vital to agricultural success, socioeconomic stability, and public health.

## 8 The challenge of Zn bioavailability and its significance

Understanding Zn bioavailability is crucial because it determines the extent to which Zn is absorbed and utilized by the body after ingestion. Bioavailability is the core of nutritional science and represents the proportion of consumed nutrients assimilated by the body and employed for physiological functions. This concept is fundamental when considering the nutritional effects of dietary Zn (Chemek et al., 2023). Although high-Zn foods are beneficial, their impact depends on the bioavailability of Zn. Animal products generally provide Zn that can be easily absorbed by the body. However, despite being Zn repositories, plant sources such as nuts and legumes might paradoxically lead to reduced Zn bioavailability because they often contain natural compounds, such as phytates, that bind Zn, making them less accessible to the body. This is particularly significant for those on vegetarian or vegan diets, who may need to consider methods for enhancing Zn absorption from plant-based foods (Trame et al., 2018; Zulfiqar et al., 2020). Soil conditions are fundamental determinants of the amount of Zn that plants absorb. Zn is readily available to plants in slightly acidic soils (Alloway, 2008). However, soil amendments such as fertilizers can affect this availability. For instance, phosphate fertilizers can reduce Zn absorption by plants, whereas organic matter in soils can bind Zn in complex molecules, making it less bioavailable to plants. Thus, management of soil health and composition is critical for maintaining adequate Zn levels in crops (Wang et al., 2019; Xie et al., 2019). The potential application of ZnO NPs to enhance crop Zn levels is an exciting development but raises questions about environmental impacts. High Zn levels can be toxic to soil organisms, which, in turn, affects soil health and plant growth (García-Gómez et al., 2020). Once absorbed by plants, Zn2+ moves through a series of complex transport pathways to where it is needed most, like seeds and fruits, which are primary food sources for humans (Broadley et al., 2007). This movement is critical for plant health and for providing Zn to those who consume these plants. Therefore, understanding and improving Zn uptake and transport within plants is vital for food security and nutrition, especially in regions where diets are predominantly plant-based, and meat consumption is low (Mosa et al., 2022; Giri et al., 2023; Ahmed et al., 2024b). An increase in Zn content and bioavailability in staple crops is crucial for addressing global nutritional needs (Zulfiqar et al., 2020). This can be achieved by breeding plants for better Zn absorption and transport, developing fertilizers to improve Zn bioavailability, and formulating dietary guidelines to optimize Zn intake (Ishfaq et al., 2023). These efforts must be environmentally sustainable in order to ensure long-term agricultural viability. Enhancing Zn bioavailability is vital not only for nutrition but also for overall global health and socio-economic stability.

## 9 Contribution of biochar in Zn bioavailability

The role of biochar in enhancing Zn bioavailability is multifaceted and has been the subject of increasing research interest, owing to its potential benefits in sustainable agriculture (**Table 1**). The carbon-rich substance produced by pyrolyzing organic matter boast a series of properties that make it advantageous for both soil health and nutrient efficiency (Shukla et al., 2023). The high surface area of biochar provides numerous adsorption sites for Zn2+ ions, making it an effective medium for Zn2+ retention in the soil. Functional groups on biochar, such as carboxyl, hydroxyl, and phenolic groups, contribute to its nutrient-binding capacity by forming complexes with metal ions, which can improve the availability of Zn2+ to plants (Tan et al., 2021). Biochar is also known for its ability to gradually release adsorbed nutrients. This controlled release mechanism is particularly valuable for Zn2+ because it ensures that nutrients are available to plants over an extended period rather than being lost through leaching or immediate reactions in the soil (Das and Ghosh, 2023). The sustained release of Zn2+ from biochar ensures that plants have a consistent and accessible supply, optimizing their Zn2+ uptake. In addition to direct nutrient interactions, biochar also affects soil biology by creating a favorable habitat for beneficial microbes, some of which play a critical role in the solubilization and mobilization of Zn2+. Certain Bacillus strains can produce organic acids, such as lactic, acetic, succinic, and formic acids, which solubilize ZnO particles in the soil, increasing the pool of available Zn2+ for plant uptake (Mumtaz et al., 2019). Furthermore, the presence of biochar has been shown to augment the effects of these microbes, particularly arbuscular mycorrhizal fungi, in increasing Zn2+ bioavailability in the soil (Vahedi et al., 2022). Additionally, some of these microbes produce siderophores and indole acetic acid, which play a role in chelating Zn2+ and promoting its uptake by plants (Mumtaz et al., 2019). The synergy between biochar and microbial activity in soils is not just theoretical but has been demonstrated in various experimental settings. For example, field studies have shown that applying biochar to soils contaminated with cadmium (Cd) can enhance wheat productivity and grain Zn content while reducing Cd levels (Farooq et al., 2020). In saline soils, where nutrient uptake can be particularly challenging, biochar has been combined with Zn-lysine complexes to improve wheat growth and physiological performance, suggesting that biochar might be part of a broader strategy to combat soil salinity issues (ul Aibdin et al., 2022). In the context of long-term soil health, biochar has been shown to increase soil fertility compared to Zn and other macronutrients. However, caution is advised to avoid potential imbalances in micronutrient availability because the high adsorptive capacity of biochar can affect the dynamics of various soil elements (Xu et al., 2022). One of the most recent advancements in the field involves the development of Zn-based nano fertilizers from carbon dots, which have been shown to significantly enhance Zn2+ bioavailability in calcareous soils, known for their challenges in nutrient availability due to high pH levels (Alikhani et al., 2023). Ultimately, biochar plays a pivotal role beyond simple soil amendment; it can be used to strategically enhance Zn2+ bioavailability in agricultural systems. When used in combination with other treatments, it shows great promise for improving nutrient uptake in crops, contributing to more robust plant growth, higher yields, and better nutritional outcomes for the food produced.

**Table 1:** Comparison of Zn content in edible portions with and without Zn NPs and biochar integration.

Crop	Control	Zn NPs	Biochar	Zn NPs + Biochar	Zn Increase	Reference
	(Zn content µg g-1 DW)				%	
Maize	14	30	–	–	114	(Adhikari et al., 2015)
Maize	41	62	–	–	51	(Choudhary et al., 2019)
Tomato	20	45	–	–	125	(Ahmed et al., 2023b)
Cherry tomato	4	5	–	–	32	(Almendros et al., 2022)
Calendula	43	–	51	–	19	(Karimi et al., 2020)
Bean	17	–	24	–	46	(Taskin et al., 2019)
Soybean	12	–	19	–	62	(Taskin et al., 2019)
Maize	18	–	36	–	100	(Taskin et al., 2019)
Lettuce	31	–	157	–	208	(Chrysargyris et al., 2020)
Rice	40	82	–	82	105	(Shukla et al., 2023)
Sunflower	7	7	–	10	43	(Seleiman et al., 2020)
Wheat	40	43	–	45	13	(Farooq et al., 2020)
Maize	37	114	–	123	232	(Rizwan et al., 2019)
Maize	35	–	–	42	20	(Zha et al., 2022)
Radish	47	–	–	95	102	(Taqdees et al., 2022)

## 10 Integration of Zn NPs with biochar: methods and techniques

The burgeoning realm of sustainable agriculture has led to the development of innovative strategies to enhance soil fertility. The integration of Zn NPs with biochar represents a testament to such endeavors, striving to capitalize on the distinct properties of both components while countering the detrimental outcomes of excessive inorganic fertilizer usage (Ali et al., 2019). This review article explores various methodologies developed for the successful amalgamation of Zn NPs with biochar, which represents an intersection of nanotechnology and agro-environmental sustainability.

**Figure 3:**
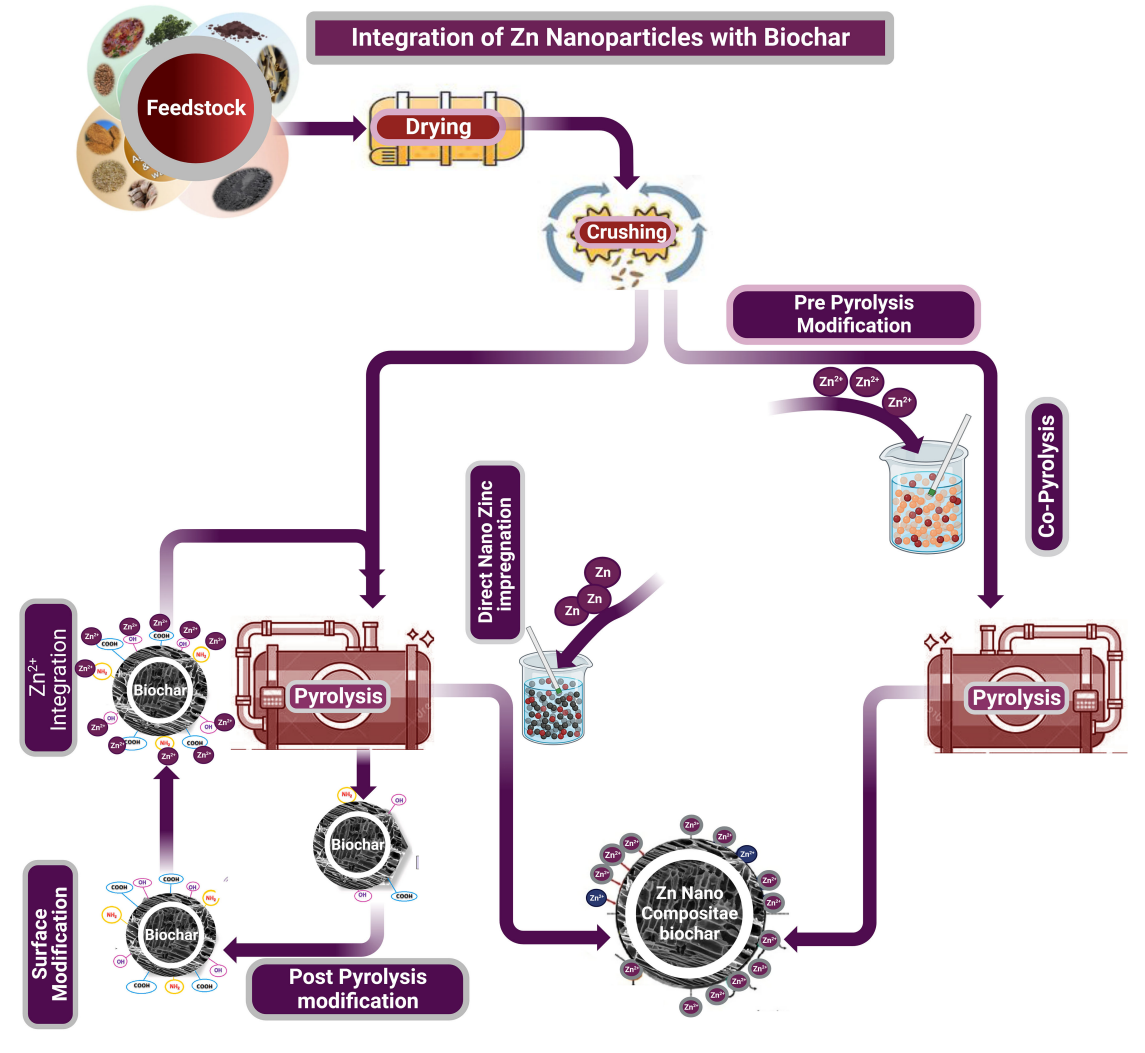
Process flow diagram for the integration of Zn NPs with biochar: This figure outlines the procedural steps for incorporating Zn NPs into biochar. It starts with feedstock preparation, followed by drying and crushing, leading into the pyrolysis process. The diagram includes both pre-pyrolysis modification, where Zn2+ ions or Zn NPs are added before pyrolysis, and post-pyrolysis modification, where Zn NPs are integrated after biochar formation. The final product is a Zn-nanocomposite biochar, designed for enhanced soil amendment and nutrient delivery in agricultural applications.

Direct impregnation involves the introduction of Zn NPs into the porous framework of biochar by using specific solvent mixtures (**Figure 3**). A colloidal solution of Zn NPs was formulated, followed by immersion in biochar, which enabled Zn NPs to pervade their intrinsic cavities. As the solvent evaporated, the biochar infused with Zn NPs remained. This method promises a homogenous distribution of Zn NPs within biochar, ensuring superior plant-root interactions when used in soils (Roy et al., 2022a).

Co-pyrolysis concurrently synthesizes Zn NPs and biochar through thermal decomposition of organic materials, including ZnO NPs (**Figure 3**). When organic matter, such as agricultural residues, is combined with a Zn source, such as ZnO, and subjected to pyrolytic conditions, high temperatures decompose the organic matter into biochar. Simultaneously, the Zn NPs became inherently embedded within the biochar structure (Jing et al., 2021).

Furthermore, biochar can be chemically modified through processes such as acid washing or alkaline treatments to improve Zn NPs adsorption (**Figure 3**), adjusting its surface for efficient nanoparticle adherence (Ahuja et al., 2022). Physical methods, particularly plasma treatments, can increase the surface roughness of biochar, offering more Zn NPs anchoring sites. Specifically, low-temperature plasma treatments with sulfur hexafluoride (SF6) gas, especially for biochar derived from starch-based packaging waste, have been introduced as sustainable modification techniques. X-ray photoelectron spectroscopy indicated that these treatments could fluorinate the biochar surface, similar to wet chemical methods, but uniquely maintained its core beneficial properties (Ahuja et al., 2022). Such modifications boost the ability of biochar to retain Zn NPs and optimize Zn delivery to plants.

Additionally, post-treatment integration is a straightforward approach; Zn NPs can be introduced into prefabricated biochar via spraying or dip-coating (**Figure 3**) (Beckinghausen et al., 2020). Although it may not guarantee a thorough Zn NP distribution, as seen in other methods, its simplicity and potential economic viability render it a suitable contender for extensive agricultural applications. The integration of Zn NPs with biochar represents an intersection of nanotechnology and sustainable agriculture. Both techniques have individual benefits and disadvantages. Therefore, selecting the best approach depends on the particular requirements of the agricultural setting and the resources at hand.

## 11 The enhanced delivery and uptake of Zn in crops through this integration

**Figure 4:**
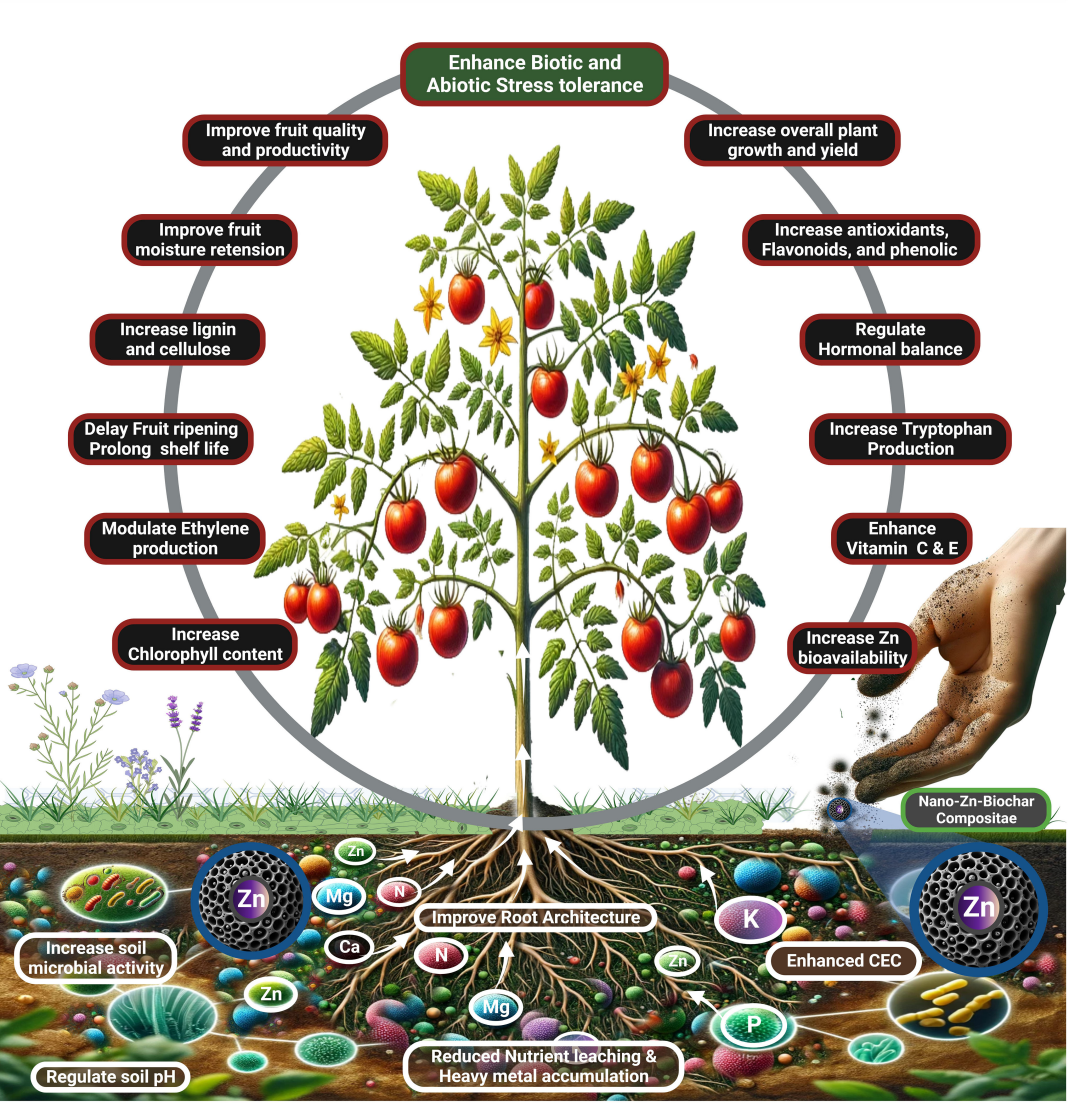
Synergistic benefits of Nano-Zn-Biochar composite on plant growth and soil health: This illustration highlights the comprehensive benefits of applying a nano-Zn-biochar composite in agriculture. It enhances plant growth and yield, improves fruit quality and shelf life, and increases essential biochemicals like chlorophyll, antioxidants, and vitamins C & E. The composite also positively impacts soil health by improving root architecture, boosting microbial activity, optimizing pH regulation, and reducing nutrient leaching and heavy metal accumulation, thereby elevating Zn bioavailability and CEC.

The dynamics of Zn in the soil, especially within the rhizosphere, is critical for effective nutrient uptake. When Zn NPs were incorporated into the biochar matrix, their porous structure served as a sustained-release nutrient source (**Figure 4**). This system efficiently addresses the issues of soil pH and competitive ion interference, which are common factors that hinder nutrient bioavailability (Baruah, 2018). Beyond enhancing micronutrient availability, the co-application of Zn NPs and biochar improves the retention and assimilation of macronutrients in the soil. The high CEC of biochar allows it to bind with key positively charged ions such as NH_4_^+^, PO_4_³⁻, and K^+^, commonly referred to as NPK nutrients. This capacity enhances nutrient retention in the soil, reduces leaching, and ensures sustained release of these essential macronutrients to plants, thereby supporting better plant growth and yield (Gwenzi et al., 2018; Das and Ghosh, 2023; Ahmed et al., 2024a). Moreover, Zn NPs influenced root morphology and exudation, thereby improving plant root nutrient uptake efficiency. This macronutrient synergy not only contributes to increased biomass but also leads to a more comprehensive nutritional profile for the resulting crops. An increase in root surface attributes is a notable benefit observed with biochar application, where studies have reported up to 58% enhancement in root development (Suganya et al., 2020). This expansive root architecture is fundamental for the uptake of water and nutrients, including Zn.

Additionally, biochar modification of soil properties can alleviate the effects of salinity on crops, thereby supporting better growth under stressful conditions (ul Aibdin et al., 2022). At the molecular level, the regulation of Zn2+ transporter genes, such as OsZIP1, OsZIP3, and OsZIP4 in rice plants offers insight into how these crops adapt to varying levels of Zn availability (Chen et al., 2008). The enhanced gene expression in response to Zn deficiency underscores the vital role of these transporters in maintaining Zn homeostasis in plants. Moreover, the presence of biochar has been linked to the decreased uptake of Cd due to the competitive inhibition of Zn2+ at the uptake sites. This is particularly important in reducing heavy metal accumulation in crops such as rice, leading to safer food production (Ali et al., 2019). In addition to nutrient dynamics, the interaction of ZnNP biochar with the soil microbiota presents another benefit. Biochar is well known for its ability to foster beneficial microbial communities, which can enhance Zn solubilization, thus amplifying its bioavailability (Vahedi et al., 2022). These microbes, along with plant-induced phytosiderophores, further boost the bioavailability and uptake of Zn, showing a remarkable efficiency in nutrient acquisition under nutrient-scarce conditions (M et al., 2013). Field applications of Zn NPs–biochar composites have consistently demonstrated positive outcomes across various crop species. Such applications have led to improved growth, higher Zn content in crops, and enhanced yields, reinforcing the potential of this strategy to transform agricultural practices (Farooq et al., 2020; ul Aibdin et al., 2022; Shukla et al., 2023). The fusion of Zn NPs with biochar has emerged as a landmark development in agricultural innovation, illuminating new pathways with the promise of fundamentally transforming sustainable farming methodologies.

## 12 Influence of Zn NP and biochar on crop growth and productivity

The challenges of Zn deficiency are evident and often cause a decline in crop production, leading to issues such as shorter internodes and stunted growth (Zagzog and Gad, 2017; Meriño-Gergichevich et al., 2021). The Zn NPs-composite has been shown to enhance root system development, which is closely linked to plant vigor. This composite provides a favorable rhizosphere environment that promotes root elongation, branching, and biomass (Baruah, 2018). Studies have noted that such improvements in root morphology are pivotal for nutrient and water absorption, ultimately influencing plant productivity (Roy et al., 2022a); Roy et al., 2022b). The enhanced CEC of biochar, combined with the solubilization properties of Zn NPs, ensures that more nutrients are available in the soil solution, leading to robust vegetative growth and increased grain or fresh weight of crops (Fazeli Sangani et al., 2020; Hafeez et al., 2022); Seleiman et al. (2020) also documented significant improvements in sunflower productivity and seed quality following the application of Zn NPs-biochar composites. The increase in seed yield, 100-seed weight, and number of seeds per head indicated the efficacy of the composite in enhancing crop yields. Zn is integral to chlorophyll production and a multitude of enzymatic reactions within plants. By increasing the bioavailability of Zn through the Zn NPs-composite, plants can perform these physiological processes more effectively, leading to improvements in growth, stress tolerance, and fruit quality (Broadley et al., 2007; Ahmed et al., 2024b). The application of Zn NPs-biochar is particularly beneficial in soils prone to Zn deficiencies, which are common in soils with high pH, low organic matter, or excessive P. These deficiencies can lead to chlorosis and stunted growth, among other issues, and the composite effectively addresses these problems (Chhabra and Kumar, 2018; Stanton et al., 2022). Adequate Zn levels ensured by the composite have shown positive effects on a wide range of crops. Improved root formation, shoot elongation, stress tolerance, and fruit set have been observed in citrus, apple, pecan, and tomato crops, which are particularly sensitive to Zn deficiency (Balafrej et al., 2020; Balandrán-Valladares et al., 2021; Mallick et al., 2021; Al Jabri et al., 2022). By addressing the underlying issues of nutrient deficiency and improving soil-plant interaction dynamics, this composite offers a promising solution for sustainable crop production.

## 13 The integration of Zn NPs with biochar for enhanced crop nutrition

The use of Zn NPs combined with biochar has emerged as a promising biofortification strategy to combat Zn deficiency in human diets by significantly enhancing Zn concentrations in crops (Ahmed et al., 2024b). The role of Zn transcends merely boosting the biosynthesis of bioactive compounds; it is integral in enhancing the nutritional value of crops by increasing the levels of vitamins, such as C and E (**Figure 4**). Such improvements are notable in health-promoting fruits such as strawberries and tomatoes, with recent research confirming the nutritional superiority of Zn-enriched produce through such methodologies (Şahin et al., 2020; Sabahat et al., 2021). The combination of biochar with Zn NPs not only enhanced the soil’s capacity for nutrient retention, but also optimized the release of these nutrients, creating a conducive environment for the uptake of essential macronutrients by plants. Consequently, crops grown in treated soils present a more comprehensive nutritional profile that contributes to dietary balance and addresses malnutrition when included in the human diet (Khan et al., 2022; Taqdees et al., 2022; Xu et al., 2022). Moreover, this treatment boosts the production of beneficial secondary metabolites in the crops. The Zn NPs-biochar synergy may encourage the synthesis of an array of phytochemicals, including antioxidants, flavonoids, and phenolic compounds, all of which are recognized for their health benefits, ranging from anti-inflammatory to anticancer properties (Ahmed et al., 2024b). This enhancement in phytochemical content positions these crops not only as nutritionally enriched, but also as carriers of additional health benefits (Almendros et al., 2022; Assunção et al., 2022). In the context of protein synthesis, Zn is essential for tryptophan biosynthesis, an amino acid crucial for plant growth hormones and seed development. The improved nutrient availability of the ZnNP-biochar composite supports crops in synthesizing proteins more efficiently, thus producing seeds and fruits with elevated protein content and a richer array of essential amino acids. Such development is particularly significant in combating protein-energy malnutrition and promoting healthy growth in the affected regions (Mosa et al., 2022; Giri et al., 2023). The strategic use of Zn NPs and biochar in agriculture not only fortifies crops with essential micronutrients, but also augments their nutritional and health-promoting qualities, thereby offering a multifaceted solution to nutritional deficiencies worldwide.

## 14 Integration of Zn NP and biochar prolonged shelf life and reduced post-harvest losses

ZnO NPs are emerging as a key innovation in agriculture, enhancing plant nutrient profiles and strengthening plant tissues to increase post-harvest freshness (**Figure 4**). These NPs reinforce plant cell walls, augmenting the synthesis of structural components, such as lignin and cellulose, which in turn boosts resilience to mechanical damage incurred during transport and storage, resulting in reduced spoilage and longer shelf life (Sharifan et al., 2021). Research has revealed that ZnO NPs coatings, particularly those based on alginate, are effective in preserving fruit quality, managing decay, and extending the shelf life of fruits like ‘Keitt’ mangoes. This is attributed to the antimicrobial properties of ZnO NPs against pathogens such as E. coli and S. aureus, which contribute to reduced post-harvest losses (Hmmam et al., 2023). One of the groundbreaking roles of ZnO NPs in agriculture is their potential to modulate ethylene production in crops post-harvest. Ethylene is a plant hormone primarily responsible for fruit ripening and senescence. Increased ethylene production can lead to faster ripening and, subsequently, a shortened shelf life (Yadav et al., 2023). As consumers demand longer-lasting fresh produce globally, ethylene management is pivotal (Ahmed et al., 2024c). Recent studies have shown that crops treated with ZnO NPs exhibit reduced ethylene synthesis, effectively delaying senescence and extending freshness. Various studies have investigated the safety of polysaccharide-ZnO nanocomposite for prolonging the shelf life of fruits. The polysaccharides used were chitosan, alginate, carrageenan, cellulose, and pectin. Overall, polysaccharide-ZnO nanocomposites can improve the shelf life of fruits by reducing weight loss, maintaining firmness, reducing ripening, respiration, and oxidation, and inhibiting microbial growth (Anugrah et al., 2020). Zn application stands out for its transformational implications in the post-harvest scenario, given its multifaceted roles in enzymatic functions and carbohydrate metabolism. Ground-breaking studies conducted by Batool et al. (2022) and Hmmam et al. (2023) validated the benefits of Zn application, shedding light on its capacity to delay the ripening of fruits such as peaches and mangoes. Additionally, Sharifan et al. (2021) reported innovative findings on the use of ZnO NPs, providing insights into the enhancement of the postharvest quality of tomatoes, showcasing the vast potential of Zn-based treatments. The use of biochar integrated with Zn NPs also aids moisture retention, further preventing dehydration and weight loss during storage (Joseph et al., 2021). This innovative approach, therefore, not only mitigates post-harvest deterioration, but also offers an added layer of food safety and nutritional improvement, showcasing the extensive potential of Zn-based interventions in the agricultural sector.

## 15 Economic benefits of combining Zn NPs with biochar

The integration of Zn NPs with biochar offers numerous economic benefits to farmers, significantly contributing to increased profitability and sustainability in agricultural practices (Akhtar et al., 2022; Roy et al., 2022a). This innovative approach not only enhances crop yields and soil health but also reduces costs associated with fertilizers and soil amendments, providing long-term economic advantages (Ye et al., 2020; Tan et al., 2022). Studies have shown that crops treated with Zn NPs and biochar exhibit better growth, higher productivity, and improved resistance to stress conditions, leading to cost savings by reducing the need for chemical fertilizers and improving nutrient-use efficiency (López-Vargas et al., 2018; Sharma et al., 2022; Das and Ghosh, 2023). The high cation CEC and porous structure of biochar improve soil aeration and water retention, creating optimal growing conditions that support robust plant development (Tusar et al., 2023; Ahmed et al., 2024a). This synergistic effect ensures that crops have access to essential nutrients throughout their growth cycles, resulting in healthier and more productive plants (Roy et al., 2022b).

One significant benefit of integrating biochar with Zn NPs is the reduction in chemical fertilizer requirements. Biochar enhances nutrient retention and reduces leaching, allowing for less frequent and lower quantities of chemical fertilizers (Gwenzi et al., 2018; Acharya et al., 2022) This reduction in fertilizer usage can lead to substantial cost savings for farmers. Additionally, biochar improves soil structure and microbial activity, further enhancing nutrient availability and reducing the dependence on external inputs (Ye et al., 2020; Alkharabsheh et al., 2021). This leads to lower input costs and mitigates the environmental impacts associated with fertilizer runoff and soil degradation (Abbas et al., 2021; Sheikhnazari et al., 2023). The long-lasting presence of biochar in the soil contributes to improved soil structure, increased microbial activity, better nutrient cycling, and enhanced soil health and productivity over time (Xu et al., 2022; Tusar et al., 2023). This sustained improvement reduced the need for soil amendments and other inputs, resulting in long-term economic benefits by maintaining high productivity levels and lower input costs. Enhanced soil health also promotes greater resilience to environmental stressors, ensuring more stable crop yields and reducing the economic risks associated with crop failure (Sui et al., 2022; Xu et al., 2022; Hasnain et al., 2023).

Biofortified crops with Zn through Zn NPs and biochar can have higher nutritional value, potentially leading to premium market prices. Nutritionally enriched produce attracts health-conscious consumers, who are willing to pay a premium (Wali et al., 2020; Ahmed et al., 2024b). For instance, Zn-enriched wheat, maize, and rice address micronutrient deficiencies in the human diet and provide market differentiation (Abbas et al., 2021; Saboor et al., 2021; Zulfiqar et al., 2021a). Moreover, this integration supports sustainable agricultural practices, reduces reliance on chemical fertilizers, and improves soil health. These practices can enhance the environmental footprint of farming operations, potentially leading to incentives or subsidies for sustainable farming from governments and organizations focused on environmental conservation (García-López et al., 2019; Rawat et al., 2019). Sustainable farming practices contribute to global efforts to combat climate change, with potential benefits from carbon credit programs or the recognition of contributions to environmental sustainability (Malik et al., 2022; Murtaza et al., 2023). In summary, the integration of Zn NPs with biochar offers significant economic benefits by boosting crop yields, reducing fertilizer costs, improving soil health, increasing market value, and supporting environmental sustainability. Continued research and adoption of this innovative strategy can lead to more resilient and profitable agricultural systems, contributing to global food security and environmental health.

## 16 Potential environmental and health concerns

The burgeoning use of Zn NPs in agriculture has been lauded for their benefits in optimizing plant nutrient uptake owing to their small size. However, they also pose potential risks, such as disrupting microbial processes such as nitrogen fixation, causing bioaccumulation in non-target organisms, and exhibiting varying effects on plant growth and soil conditions (Hazeem, 2022; Shah et al., 2022; Lahive et al., 2023), necessitating comprehensive environmental risk assessments to protect soil biodiversity and ensure sustainable agriculture (**Table 2**). The incorporation of Zn NPs into soil can disrupt essential microbial processes, potentially hindering N-fixation, as evidenced by the negative impact on the N-fixing area of root nodules and bacteroids in alfalfa, indicating a reduction in N-fixing capability and organic matter decomposition (Shah et al., 2022). Moreover, the persistence of Zn NPs in the soil could harm non-target organisms. For instance, earthworms in ZnO NP-laden soils show higher Zn tissue concentrations, raising concerns regarding the bioaccumulation and trophic transfer of Zn in terrestrial ecosystems (Lahive et al., 2023).

**Table 2:** Adverse effects of ZN NPs on plant growth and soil health across various crops.

Zn NPs Concentration	Crop Type	Adverse effect on plant growth, soil health and microbial activity	References
100–200 mg L-1	Wheat and Maize	While ZnO NPs up to 100 mg/L benefit plant growth, concentrations over 150 mg/L cause toxicity, harming plant health and metabolism.	(Srivastav et al., 2021)
100-1000 mg kg-1	–	ZnO NPs reduced microbial biomass carbon by 27.0-33.5% at 100 mg/kg and by 39.0-43.3% at 1000 mg/kg, irrespective of biochar addition.	(Shemawar et al., 2021)
1000 mg kg-1	Spinach	ZnONPs in manure-treated soils decreased microbial activity, reduced bacterial and fungal colonies, lowered soil mineral N content, and diminished spinach yield and plant N recovery.	(Shah et al., 2022)
100, 1000, and 10,000 mg kg-1	Radish	Cu and Zn NPs showed high ecotoxicity, reducing bacteria and Azotobacter sp. abundance, and radish seed germination and root length more than enzymatic activities	(Kolesnikov et al., 2021)
ZnO NPs at 500 mg kg-1 and Cd at 10 mg kg-1	American pokeweed	ZnONPs and Cd exposure inhibited root growth by 43%, increased Cd in shoots, and caused severe root cell damage.	(Xiao et al., 2022)
ZnONPs 0–500 mg kg-1	Soybean	Lower concentrations (up to 200 mg/kg) of both ZnONPs and Zn²^+^ ions benefited root growth and development. However, concentrations above 200 mg/kg inhibited root growth and overall plant development, and caused soil pH and electrical conductivity imbalances, potentially affecting soil health and microbial activity.	(Yusefi-Tanha et al., 2022)
Zn nano and bulk 0, 25, 100 mg kg-1	Rice	Low concentrations of FeO and ZnO NPs enhanced rice growth and grain quality but altered the soil microbial community by reducing key phyla such as Proteobacteria, Actinobacteria, and Planctomycetes, which impacted essential soil functions like chitin degradation, ammonia oxidation, and nitrite reduction.	(Afzal and Singh, 2022)
200 mgL-1	Wheat and Tomato	Compared to bulk ZnO, nZn and nZnO exhibited higher toxicity, affecting seed germination, growth parameters, chlorophyll, and carotenoid contents, and leading to increased Zn bioaccumulation. nZnO often caused more adverse effects than nZn at similar concentrations. Zn accumulation was highest in Zn2+ ions, followed by nZn, nZnO, bulk ZnO, and the control. Exposure to 200 mg Zn·L−1 of nZn and nZnO elevated H2O2 and MDA levels, especially in tomatoes, indicating greater oxidative stress compared to wheat.	(Amooaghaie et al., 2017)
ZnO NPs and Zn sulfate (ZnSO4) 0-400 mg Kg-1	Coriander	At 400 mg kg-1 of ZnSO4, there was a significant 34.6% decrease in chlorophyll content. This exposure led to increased Zn absorption in both roots and shoots, resulting in elevated levels of H2O2 and MDA, which are indicators of oxidative stress. The observed toxicity was primarily attributed to the accumulation of dissolved Zn2+ ions in plant tissues.	(Ruiz-Torres et al., 2021)

ZnO NPs can accumulate in organisms, potentially leading to bioaccumulation in the food chain and other adverse ecological effects. Although higher ZnONP levels can impede seedling growth by inducing oxidative stress, a concentration of 20 ppm enhanced growth and nutrient content in mung bean seedlings, suggesting that it is a viable concentration for biofortification without detrimental effects (Sorahinobar et al., 2023). The varying effects of Zn NPs based on soil acidity underscores the need for comprehensive environmental risk assessments to protect soil biodiversity and promote sustainable agriculture (García-Gómez et al., 2020). The interactions between biochar and Zn NPs are also a critical area of study. The properties of biochar can vary widely depending on the feedstock and pyrolysis conditions, influencing its potential to adsorb Zn NPs and mitigate or exacerbate their environmental impacts (Jian et al., 2018; Tomczyk et al., 2020; Das et al., 2021). Therefore, selection of an appropriate biochar for specific applications is essential to prevent adverse environmental outcomes.

On the human health front, while Zn is a vital nutrient, excessive accumulation in crops treated with Zn NPs could lead to Zn overdose, with symptoms ranging from stomach cramps to more severe conditions such as anemia or neurodegenerative diseases (Plum et al., 2010; Dhaliwal et al., 2022). The role of Zn in apoptosis and cell death, particularly brain injury, highlights the complexity of its biological impact and the delicate balance required for homeostatic regulation (Plum et al., 2010). Furthermore, excessive Zn intake in children can result in obesity and related health issues (Arfi et al., 2021). The environmental footprint of Zn NPs and biochar extends beyond the terrestrial boundaries. There is a risk of leaching these substances into groundwater, which may lead to toxicity in aquatic ecosystems and contamination of potable water supplies (Westerhoff et al., 2018; Hazeem, 2022). The potential of biochar to reduce this leaching through adsorption requires more clarity to understand its long-term ecological interactions.

Navigating the use of Zn NPs in agriculture requires a careful assessment of their benefits in boosting plant health and growth, as seen with the lower concentration for crop biofortification (Sorahinobar et al., 2023), against potential risks to ecosystems and human well-being. Striking a responsible balance demands rigorous research into mitigating strategies and refinement of usage guidelines, aiming to achieve a harmonious integration of Zn NPs into farming practices that are both productive and protective of our natural and human environments.

## 17 Final considerations and future directions

The synergy between Zn NPs and biochar has the potential to revolutionize sustainable agricultural practices. However, the key lies in determining correct balance. Although both components can individually contribute to plant health, their combined efficacy depends on their ratios. It is crucial to identify optimal ratios for different crop types, soil compositions, and climatic conditions. For instance, sandy soils might require different Zn NPs-biochar proportions than clayey soils. Experimental studies, both in controlled environments and field trials, can provide insights into these specifics. Once optimized, these ratios can help achieve maximum crop productivity, nutrient density, and stress resistance while ensuring environmental and health safety (Hasnain et al., 2023).

Many studies on Zn NPs and biochar have been conducted under controlled conditions or in short-term field trials. Understanding the long-term implications of integrating these components in agriculture is of paramount importance. Future research should focus on extended field trials spanning multiple crop cycles to reveal cumulative benefits or challenges, such as changes in soil structure, microbial communities, and long-term crop health. Comparative studies across different crops and climatic regions will provide valuable insight into the versatility and robustness of this approach. Such trials can also uncover unforeseen interactions between Zn NPs, biochar, and other agricultural inputs such as fertilizers and pesticides (Vijay et al., 2021; Gupta et al., 2022).

The benefits of integrating Zn NPs with biochar are apparent, but the underlying molecular and physiological mechanisms remain unclear. Inconsistencies in current studies, particularly regarding long-term stability and environmental impact, need to be addressed. Recognizing specific gene expression or metabolic pathways that are positively impacted can aid in breeding or engineering crops that are more responsive to Zn NPs-biochar treatments. Future research should focus on long-term field trials to evaluate the sustainability and cumulative benefits or challenges of ZnNP-biochar integration. Techniques such as transcriptomics, proteomics, and metabolomics provide a comprehensive view of these interactions and their implications for plant health (Seleiman et al., 2020; Kumar et al., 2021). As with any novel agricultural intervention, the introduction of Zn NPs and biochar on a large scale requires clear regulations and policies. Policymakers can leverage the insights from scientific research, long-term field trials, and stakeholder feedback to draft more comprehensive regulations. Additionally, international collaboration can help harmonize these guidelines and ensure consistency and safety across borders.

Combining Zn NPs with biochar has emerged as a potential strategy for sustainable agriculture, enhancing plant growth, and addressing human micronutrient deficiencies. This dual-action approach improves soil health, aids nutrient absorption, and boosts plant resilience against environmental stressors, thereby increasing yields. Zn NPs are crucial for protein synthesis and enzyme activity, fortifying plants against various stressors. Biofortification of crops such as maize, tomato, lettuce, and rice with Zn has been successful in enriching the Zn content and reducing global micronutrient malnutrition. Innovative cultivation methods, such as nutrient priming with ZnSO4 or Zn-EDTA, further support these findings. However, the introduction of Zn NPs poses environmental and health risks, including potential biomagnification and the disruption of soil microbial processes. Therefore, although the benefits for crop productivity and nutrition are significant, a cautious approach is necessary. Optimizing the use of Zn NPs and biochar, along with rigorous investigation of their potential drawbacks, is crucial. Future research should focus on the development of sustainable application methods to ensure safety and efficacy. In conclusion, integrating Zn NPs with biochar offers a promising solution for enhancing crop yield and nutritional quality while maintaining environmental sustainability. This strategy can significantly impact food security and public health; however, comprehensive risk assessments and best practices are essential to mitigate the associated risks.

## Author’s note

During the preparation of this work, the authors used Paperpal to check grammar, sentence structure, and punctuation. After using this tool/service, the authors reviewed and edited the content as required and took full responsibility for the content of the publication.
